# Supplementary material for: Heterologous Expression and Biochemical Characterization of a Novel Lytic Polysaccharide Monooxygenase from Chitinilyticum aquatile CSC-1
Source: Microorganisms. 2024 Jul 8;12(7):1381. doi: 10.3390/microorganisms12071381 (PMC11278713; doi:10.3390/microorganisms12071381)
Supplement: Supplementary file 1 [file microorganisms-12-01381-s001.zip › microorganisms-3069460-supplementary.pdf]

Supplementary Material

Heterologous expression and biochemical characterization of a novel lytic polysaccharide monooxygenase from *Chitinilyticum aquatile* CSC-1

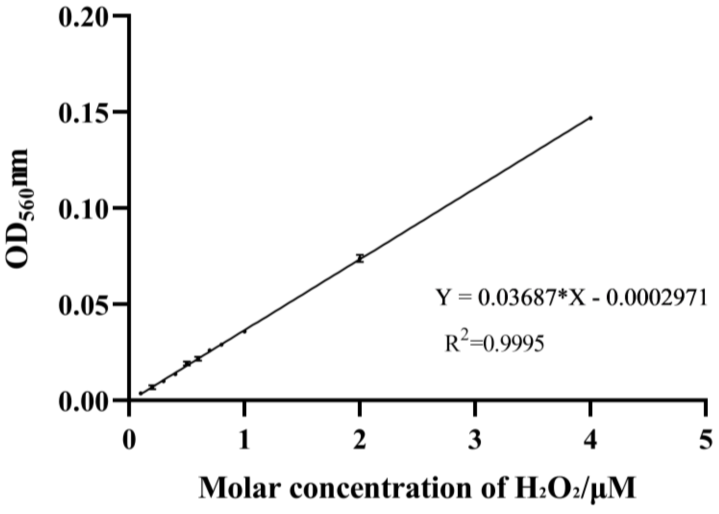

Supplementary Figure S1. H_2O_2 standard curve

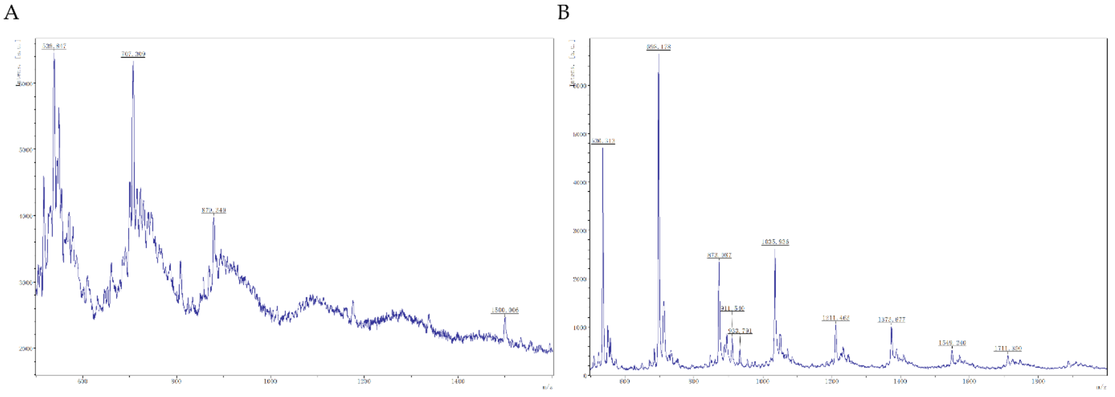

Supplementary Figure S2. Mass spectrum of the production of cellulose and chitin in the absence of *Ca*LPMO10. A: Mass spectra of products without *Ca*LPMO10, featuring only ascorbic acid and cellulose; B: Mass spectra of products without *Ca*LPMO10, featuring only ascorbic acid and chitin

Table S1 The specific primers

Primers	Nucleotide sequence(5'-3')
CaLPMO10-F	5'- cccagccggcgatggccatggCCCACGGCACCATGGCTG -3'
CaLPMO10-R	5'- gtggtggtggtggtgctcgagTTTGCAGGTGCCTAGGTCCTT -3'
